# Biothermodynamics of Viruses from Absolute Zero (1950) to Virothermodynamics (2022)

**DOI:** 10.3390/vaccines10122112

**Published:** 2022-12-09

**Authors:** Marko Popovic

**Affiliations:** School of Life Sciences, Technical University of Munich, 85354 Freising, Germany; marko.popovic@tum.de or marko.popovic.td@gmail.com

**Keywords:** thermodynamics, calorimetry, entropy, enthalpy, Gibbs energy, virus–host interaction, SARS-CoV-2, COVID-19, Ebola virus

## Abstract

Biothermodynamics of viruses is among the youngest but most rapidly developing scientific disciplines. During the COVID-19 pandemic, it closely followed the results published by molecular biologists. Empirical formulas were published for 50 viruses and thermodynamic properties for multiple viruses and virus variants, including all variants of concern of SARS-CoV-2, SARS-CoV, MERS-CoV, Ebola virus, Vaccinia and Monkeypox virus. A review of the development of biothermodynamics of viruses during the last several decades and intense development during the last 3 years is described in this paper.

## 1. Introduction

### 1.1. From Thermodynamics to Biothermodynamics

There is a common opinion that thermodynamics is a scientific discipline related to machines, engines and devices, dealing mostly with efficiency of energy transformation and utilization. Indeed, Lazarus Carnot [1,2] and his son Sadi Carnot [3] have, through their brilliant research, imposed such a perception into the public for over two centuries [4]. In this way, classical thermodynamics began its development. It is less widely known that, simultaneously with classical thermodynamics, appeared biothermodynamics. Lavoisier and Laplace [5,6] developed the first calorimeter and one of the first samples for calorimetry was an organism—a live guinea pig. Thus, simultaneously with classical thermodynamics, biothermodynamics started its development.

Often, the same researchers worked in the field of classical thermodynamics and biothermodynamics. Indeed, Boltzmann [7], one of the founders of statistical thermodynamics, has written about change in entropy in living organisms. Clausius [8,9,10] has laid the theoretical foundations of classical thermodynamics, with the goal of analyzing machines. However, von Bertalanffy [11] has suggested the theory of open systems in biology. Schrödinger in his famous book “What is Life?” discussed the thermodynamic background of life processes [12]. Morowitz [13,14,15] has discussed potential controversies related to self-assembly in organisms and emergence of life, and the second law of thermodynamics.

Growth is one of the main characteristics of organisms. The answer to the question of what represents the driving force for the growth of organisms was given by von Stockar [16,17,18,19,20]. It seems that biothermodynamics, even though it is less widely known than classical thermodynamics, has existed in the scientific arena for as long, and has given impressive results. Hansen analyzed whether an extended thermodynamic framework can be used to analyze processes in organisms that involve information, such as biological evolution [21,22,23]. Application of thermodynamics to biological evolution was also discussed by Skene [24]. Battley has made a great contribution towards applying the quantitative thermodynamic approach to living organisms and life processes [25,26,27,28,29,30]. Roels [16,31], and Sandler [32,33] have also contributed to quantifying the thermodynamic properties of organisms. Barros has applied thermodynamics to study the growth of microorganisms in soil ecosystems [34,35,36]. Maskow has applied calorimetry and thermodynamic analysis to study the growth of microorganisms in bioreactors [37,38] and ecosystems [39,40], as well as viruses in host cells [41]. Guosheng et al. [42] have also applied calorimetric methods to study the multiplication of bacteriophages inside host cells.

### 1.2. Biothermodynamics Intersects with Biochemistry

Thermodynamic characterization of life processes has been a subject of interest for many researchers. Von Stockar et al. [19,43] applied thermodynamics to quantitatively analyze thermodynamic feasibility of complex metabolic pathways, such as glycolysis. Thermodynamic analysis has been used to find accurate Gibbs energy values with activity coefficient corrections for important biological reactions, including Hexokinase reaction [44], Glucose-6-phosphatase reaction and ATP hydrolysis [45], 3-phosphoglycerate kinase reaction [46], Triosephosphate isomerase reaction [47], Enolase reaction [48], and Glyceraldehyde 3-phosphate dehydrogenase reaction [49]. Additionally, thermodynamic analysis was made of cellulose hydrolysis by microorganisms in the aqueous glucose solution [50]. Niebel et al. [51] found that the cellular metabolism is governed by an upper limit in Gibbs energy dissipation, using metabolomics. Ould-Moulaye et al. [52] found Gibbs energy changes for the reactions in glycolysis and Krebbs cycle. Kümmel et al. [53] discuss applications of thermodynamics in metabolic network models.

The importance of thermodynamic considerations in life sciences is clearly seen from the Gibbs energy being used to define catabolic and anabolic processes [54]. Annamalai used the quantitative thermodynamic approach to study the metabolic processes [55,56] and the aging of organisms [57,58,59,60,61]. Hayflick was among the first who related a thermodynamic property (entropy) to the aging process in a series of papers [62,63,64,65,66,67,68,69].

### 1.3. From Biothermodynamics to Virothermodynamics

Viruses are the most abundant organisms: there could be more viruses than stars in the universe [70]. There are 9,110 named species listed by the International Committee on Taxonomy of Viruses (ICTV) [71]. Until 2019, despite the wide variety of viruses, they have been the subject of research of microbiology, virology, biology and medicine. However, inside host cells, viruses represent growing open chemical and thermodynamic systems [72,73,74,75]. Until 2019, elemental composition was known only for the poliovirus [76,77]. This is a consequence of the fact that analytical laboratories rarely have biosafety levels required for work with most viruses, as well as the fact that viruses are difficult to isolate in sufficient amounts and purity [78]. Until recently, viruses were not a subject of thermodynamic research. The thermodynamic properties of virus particles and nucleocapsids were unknown.

With the appearance of the COVID-19 pandemic, various scientific disciplines attempted to contribute, in the shortest time possible, to the fight against the pandemic. Molecular biology has played an important role with the reading of genetic sequences of SARS-CoV-2. Thermodynamics has joined the fight and in 2020, thermodynamic properties have been published for multiple viruses [79]. An analysis was made of virus–host interactions in the cytoplasm (virus multiplication) [79]. The first empirical formula and thermodynamic properties of the Hu-1 variant of SARS-CoV-2, as well as SARS-CoV and MERS-CoV were published in 2020 [80]. In 2020, in parallel with the COVID-19 pandemic, an epidemic caused by the rhinovirus occurred, while the influenza epidemic did not occur that year. An explanation of coinfection by rhinovirus and SARS-CoV-2, and interference between influenza and SARS-CoV-2 has been published in [81]. SARS-CoV-2 belongs to the group of RNA viruses, which exhibit a great tendency to mutate [82]. Thus, during the 2.5 years of the pandemic, the virus has mutated several times [83,84,85,86]. The mutants suppressed the older variants and caused new waves of infection during the pandemic. The elemental composition and thermodynamic properties of SARS-CoV-2 variants from Hu-1 to Omicron BA.2.75 have been published in [80,86,87,88,89,90,91,92,93]. The biothermodynamic characterization of viruses was continued for Monkeypox, Vaccinia and Ebola viruses [94,95].

Infectivity and pathogenicity are terms mostly used in microbiology, biology and medicine. These terms have their physical basis and driving forces in biothermodynamics. The basis of the infectivity of viruses is susceptibility and permissiveness (binding affinity and multiplication rate, respectively). Antigen–receptor binding represents a chemical reaction, similar to protein–ligand interactions [96]. The driving force for antigen–receptor binding is the Gibbs energy of binding [86,88,91,97,98,99,100,101]. Thus, biothermodynamic consideration and determination of Gibbs energy of binding is very important for infection spreading [102,103]. More negative Gibbs energy of binding of new variants gave an advantage to new strains during entry over older ones, which led to faster spreading of the virus and shorter incubation period. Gibbs energies of binding and binding affinities of viruses have been reported in the literature for various viruses [86,87,88,89,90,91,95,97,98,99,100,101,104].

To explore the interaction between a virus and its human host, it was necessary to find thermodynamic properties for host organisms. Thermodynamic properties have been reported for human tissues [95,105] since virus–human interactions have been of particular importance. Thermodynamic properties of plant host organisms are reported in [106]. Phage–bacteria interactions are often used as a model in the research of virus–host interactions. Thus, thermodynamic properties have been determined for a large number of bacteria [29,107,108,109,110] and bacteriophages [41,42,79].

The second virus–host interaction is in the cytoplasm. In papers [79,80], a biothermodynamic mechanism was suggested for virus hijacking of host cell metabolism. The permissiveness represents the ability of a virus to multiply inside the host [111]. The multiplication of a virus represents a chemical reaction of polymerization of nucleotides into nucleic acids, and amino acids into structural and functional proteins of the virus [95]. The driving force for these reactions is the Gibbs energy of biosynthesis [112]. After their biosynthesis, the virus components undergo self-assembly into a new virus particle [113,114]. During biosynthesis and self-assembly, viruses change their thermodynamic properties [115,116]. Thus, the virus life cycle represents a biological, chemical and thermodynamic process that should be analyzed using a nonequilibrium thermodynamic apparatus [117].

Viruses represent the smallest organisms, but also belong to the most contagious and deadly microorganisms. They spread very rapidly, often causing epidemics and pandemics, which result in large numbers of casualties. Furthermore, there are very few antiviral medicines. Thus, the fight against epidemics and pandemics is directed towards epidemiological measures and the application of vaccines. However, vaccine production, especially in the case of new viruses, requires a lot of time and resources. For example, the vaccines against SARS-CoV-2 were awaited for a year. The ability of some viruses to develop mutations fast leads to the need for new vaccines. Some of the available novel vaccines have proved themselves effective for the Hu-1, Alpha, Beta, Gamma and Delta variants. However, these vaccines are much less effective for the newer Omicron variants due to their ability to evade the immune response. This has imposed a need for the production of polyvalent vaccines, which also takes time and long-term testing. Knowing the thermodynamic properties of the host and virus, as well as the application of a mechanistic model of interactions on the cell membrane and in the cytoplasm, could, in the future, contribute to designing new vaccines and antiviral medicines. Moreover, such knowledge could aid in finding places and methods for vaccine application. For example, every human tissue is characterized by a specific value of Gibbs energy of biosynthesis of its building blocks. On the other hand, every virus variant is characterized with its own specific Gibbs energy of biosynthesis. The ratio of these two values is the permissiveness coefficient, which is different for various virus–host cell pairs. The result of this is that some viruses can be synthetized in one type of cell, while in others their multiplication is significantly slower. By choosing a tissue for vaccine application where virus growth is slower, it is possible to give enough time to the immune system to respond to a low virus concentration. Such a vaccine would be attenuated (live), capable of inducing an immune response but, due to the low permissiveness coefficient, unable to cause a disease in a more severe clinical form. The attenuation process of a vaccine based on biothermodynamic properties would not be performed through a long passage that requires great resources and time, but through choosing a place of application where the virus can multiply very slowly. Thus, one of the potential applications of biothermodynamics in virology would be in vaccinology. Such a vaccine would not be based on empirical data but on engineering, using biothermodynamic tools, which would help to significantly save time and resources in the design and production of vaccines.

The aim of this review paper is to summarize the intense development of viruses in the field of biothermodynamics during the last few decades and try to predict the directions of the future development of the youngest scientific discipline—virothermodynamics.

## 2. Methods and Results

This section discusses the methodologies used in biothermodynamics of viruses and the results they provide. First, the experimental techniques are discussed, followed by theoretical approaches.

### 2.1. Experimental Approaches in Biothermodynamics of Viruses

The binding affinities of virus antigens to host cell receptors (susceptibility) have been measured using surface plasmon resonance [118,119] and the non-competitive ELISA approach [120,121]. Surface plasmon resonance (SPR) gives kinetic and thermodynamic data on antigen–receptor binding, including association rate constant, *k_on_*, dissociation rate constant, *k_off_*, and dissociation equilibrium constant, *K_D_* [118,119]. SPR is a label-free optical technique that measures biomolecular interactions in real time by detecting reflected light from a prism-gold film interface [118]. The non-competitive ELISA approach measures the thermodynamic properties of antigen–receptor binding [120,121]. It represents a simple, rapid, and reliable method for measuring dissociation equilibrium constants, *K_D_* [120,121]. The experimental results can be used to calculate other important parameters of antigen–receptor binding, including binding equilibrium constants, *K_B_*, standard Gibbs energies of binding, Δ*_B_G⁰*, binding phenomenological coefficients, *L_B_*, and binding rates, *r_B_* [88,91].

Calorimetry has been used to study viruses, including differential scanning calorimetry (DSC), isothermal titration calorimetry (ITC) and reaction calorimetry (isothermal microcalorimetry). Differential scanning calorimetry (DSC) measures the difference in heat absorption rates between sample and reference during gradual heating, revealing various thermal effects, such as phase transitions or protein unfolding [122,123]. DSC has been used since the 1970s in research on viruses, including measurements of energetics of virus capsid self-assembly and denaturation [124,125], virus particle structure [126,127], thermal stability [125,128,129,130], virus identification [124], virus denaturation [131,132], entry into host cell [133,134], capsid self-assembly [135,136] and vaccine development [137,138].

While DSC performs measurements by changing temperature, isothermal titration calorimetry (ITC) measures heat released or absorbed when a reagent is titrated into a solution at constant temperature [122,123]. ITC was also applied to study a wide range of phenomena related to viruses, such as virus adsorption and disassembly [139], influence on metabolism and cell cycle [140,141], apoptosis inhibition [142,143], virus structure and entry into host cells [144], nucleocapsid self-assembly [145], inactivation [146,147], immune response evasion [148], antiviral therapy development [149,150,151,152], vaccine development [153], etc.

Reaction calorimetry, or isothermal microcalorimetry, measures heat released or absorbed during a chemical reaction, usually at constant temperature (without titration like in ITC) [122,123]. Reaction calorimetry has been applied to study virus multiplication inside host cells [42,154,155,156], phage action against bacterial biofilms [155,157,158,159,160,161,162], phage-bacteria interactions [163,164], phage transition from lytic into lysogenic cycles [41], antiviral and phage therapy [165,166,167], and influence on marine ecosystem metabolism [168].

### 2.2. Theoretical Approaches in Biothermodynamics of Viruses

The thermodynamic properties of viruses can be calculated using biothermodynamic methodology. Thermodynamic properties of virus biosynthesis can be found from virus elemental composition in three steps:

(1) Empirical formula;
(2) Thermodynamic properties of live matter;
(3) Thermodynamic properties of biosynthesis.

The first step is to find empirical formulas of virus live matter. This can be achieved using the atom-counting method [78], which gives elemental composition of viruses using widely available data on genetic sequences [169,170,171,172,173], protein sequences [169,170,174] and virus morphology [78]. The second step is to calculate thermodynamic properties of virus live matter, using predictive biothermodynamic models [78]. Elemental composition of virus live matter can be used to find its thermodynamic properties using the Patel–Erickson equation [28,88,107,175], Battley equation [26,88,107] and Hurst–Harrison equation [176,177]. The third step is to use elemental composition of live matter to construct biosynthesis reactions for the viruses [79,88]. The biosynthesis reactions are combined with thermodynamic properties of live matter to find thermodynamic properties of biosynthesis [79,88].

Phenomenological equations are an important tool, relating thermodynamic and kinetic properties of processes [17,178,179]. Phenomenological equations are intuitive and simple to apply, stating that the rate of a process is proportional to its thermodynamic driving force—Gibbs energy [178,179]. A phenomenological equation for a chemical process has the general form [178,179]

(1) $$r=-\frac{L}{T}\Delta G$$

where *r* is the rate of a chemical process, *T* is temperature, while Δ*G* is Gibbs energy change of the process. *L* is a constant known as phenomenological coefficient, and is specific for each process. Phenomenological equations can be applied to both antigen–receptor binding and virus multiplication inside host cells [88,91]. In the case of antigen–receptor binding, the binding phenomenological equation relates binding rate, *r_B_*, and Gibbs energy of binding, Δ*_B_G*:

(2) $$r_{B}=-\frac{L_{B}}{T}\Delta_{B}G$$

where *L_B_* is the binding phenomenological coefficient [88,91,180].

Similarly, the biosynthesis phenomenological equation relates the rate of biosynthesis of virus components, *r_bs_*, to the Gibbs energy of biosynthesis, Δ*_bs_G*:

(3) $$r_{bs}=-\frac{L_{bs}}{T}\Delta_{bs}G$$

where *L_bs_* is the biosynthesis phenomenological coefficient [88,95,180]. Phenomenological equations have also been applied to analyze growth of bacteria [17,178].

Experimental work with viruses can sometimes require high biosafety levels. However, there are few laboratories that work on calorimetric measurements that possess the appropriate biosafety level [78]. Time, especially in circumstances of epidemics/pandemics caused by dangerous viruses, plays a very important role in suppressing infections. Thus, computational methods (especially since the beginning of the COVID-19 pandemic) have been gaining in importance [181], since they have proved themselves to be a fast and accurate source of information on kinetics and the biothermodynamic background of virus–host interactions. Three-dimensional-QSAR modeling is effective for predicting novel inhibitors from an existing scaffold and defining the influence of chemical properties on bioactivities [181]. Combinatorial molecular docking provides active site conformational details, while the inclusion of other dynamical methods would improve predictive capability [181]. Molecular docking can be used to predict binding affinities [181]. Machine learning algorithms have been used for the research of virus–host interactions, including immune responses [182]. Molecular dynamics and 3D-QSAR have been used to study binding mechanisms of the Hepatitis B virus [183]. Computational approaches, such as docking, have been applied extensively to study protein–protein interactions, since experimental data is often limited [184]. Computational approaches have been used to characterize SARS-CoV-2 variants of concern, including the effect of mutations on the binding affinity of the receptor-binding domain (RBD) to human angiotensin-converting enzyme 2 (hACE2) [185,186]. Moreover, computational approaches have been used to identify antibodies that neutralize SARS-CoV-2 and other virus particles [187,188]. All these methods give useful information that can be, using biothermodynamic methodology, applied for finding the driving force for antigen–receptor binding—Gibbs energy.

## 3. Discussion

The path from thermodynamics to biothermodynamics was very short. The researchers who laid the foundations of classical thermodynamics were also the first to apply them to living organisms [5,6]. The road from biothermodynamics to biothermodynamics of viruses, virothermodynamics, has been much longer. It lasted 150 years. In that period, the basis was laid for experimental measurements on virus samples, as well as the methodology for theoretical analysis. Thus, the opportunities for virus research offered by biothermodynamics are great. However, the limiting factor for research represents the problem of providing biological samples of sufficient size and adequate purity, high sample prices, as well as finding laboratories with the required biosafety level and personnel ready to work on biothermodynamics [78]. Having in mind that the discipline is really young, biothermodynamics courses are rarely offered at universities in Europe, even though it seems that students are showing interest for this discipline. In this early period of development of biothermodynamics of viruses, of particular importance are results of molecular biology, which have made the data on sequences of nucleic acids and proteins widely available, as well as the work of virologists who made available data on virus morphology.

In the introduction, it was mentioned that viruses represent the most abundant living organisms. Moreover, there is nearly 10 000 different virus species. However, during the last few years, empirical formulas have been determined for less than 50 species, while thermodynamic properties are known for less than 70 species. Various virus species (and variants) are characterized by specific empirical formulas. For example, the Hu-1 variant (wild type) of SARS-CoV-2 is characterized by its specific empirical formula CH_1.6390_O_0.2851_N_0.2301_P_0.0065_S_0.0038_ [80,112]. The empirical formula of the Ebola virus is CH_1.569_O_0.3281_N_0.2786_P_0.00173_S_0.00258_ [95]. This difference in empirical formulas can be used for the identification of various virus species and their variants, using single particle inductively coupled plasma mass spectroscopy analysis (SP-ICP-MS) [93] or the atom-counting method [78]. Moreover, each variant of SARS-CoV-2 is characterized by its own empirical formula [80,88,89,112].

Panta rhei; the world is moving and changing. The natural driving forces are hidden in the objective world and the human body. What are the physicochemical forces that drive life? Organisms perform biological and chemical processes. The driving force of all chemical processes in animate and inanimate matter is Gibbs energy [17,178,179,189,190]. This is why Gibbs energy represents the driving force for interactions of organisms with their environment [16,17,18,20,191].

Viruses represent obligate intracellular parasites [192]. Thus, the environment of viruses during their life cycle is animate matter—host cell. Therefore, the virus interacts with its host cell at the membrane, by binding to specific receptors on the host cell surface [193] and entry of the host cells, as well as inside the cell in the cytoplasm, performing replication, transcription, translation, self-assembly and maturation. After maturation, new virions leave the cell, leading to its damage. All these phenomena represent chemical reactions or physical processes. Antigen–receptor binding represents a chemical reaction similar to protein–ligand interactions [91,96]. Transcription represents the process of transmission of information, based on polymerization of nucleotides into RNA [194]. Translation represents a process of conversion of information from the RNA code into a protein code, based on a polymerization reaction of amino acids [195]. Both these process, as well as replication of nucleic acids [196,197] are driven by Gibbs energy of biosynthesis. Biosynthesis reaction of structural and functional proteins of cells and biosynthesis of virus components are competitive. According to equations (1) and (3), reaction rate depends on Gibbs energy of biosynthesis. During competition, the reaction that occurs faster has an advantage. This is the way in which a virus hijacks the host cell’s metabolism. In order to predict the outcome of this interaction, it is necessary to know thermodynamic properties (Gibbs energy, entropy and enthalpy) of both the virus and its host cell.

The permissiveness coefficient represents the ratio of rates of biosynthesis of virus components and host cell components. A permissiveness coefficient greater than one indicates the advantage in synthesis of virus components, leading to a successful viral life cycle inside the host. The permissiveness coefficient, *P*, is given by the equation

(4) $$P=\frac{r_{bs}\left(virus\right)}{r_{bs}\left(host\right)}=\frac{\Delta_{bs}G^{0}\left(virus\right)}{\Delta_{bs}G^{0}\left(host\right)}$$

where *r_bs_* represents the biosynthesis rate, while Δ*_bs_G⁰* is standard Gibbs energy of biosynthesis [95]. A similar method is used in pharmacology, in research on the interactions of two medicines (e.g., synergistic, antagonistic, or neutral interactions), during simultaneous application on cells [198]. Basically, interaction between the medicine and the cell, and the virus with its cell represent the same process, similar to protein–ligand interactions. Thus, there is a similar approach in pharmacology and biothermodynamics. Since the processes are similar, they obey the same chemical and biothermodynamic laws, hence the similarity in approach and applied equations. By comparing permissiveness coefficients for two different viruses (or virus variants) for the same host tissue, it is possible to conclude whether there will be coinfection or interference during simultaneous contact with both viruses by the same host. This practical application can be of use to epidemiologists and infectologists since it is not rare for two viruses to appear in the same population at the same time and in the same place. If permissiveness coefficients of two viruses are similar for the same tissue, then the probabilities of virus multiplication will be similar. Such was the case with SARS-CoV-2 and rhinovirus [81]. This resulted in the simultaneous occurrence of COVID-19 pandemic and an epidemic caused by the rhinovirus. Additionally, a similar observation was made with epidemics caused by influenza and parainfluenza viruses. On the other hand, if there is a significant difference in permissiveness coefficients between two potential causes of epidemics, then one epidemic will suppress the other. This happened in the winter of 2020/21 and 2021/22, when the influenza epidemics did not occur during the COVID-19 pandemic [81].

It is obvious that the biothermodynamics of viruses are able to offer a wide variety of important information, useful first of all to virologists, microbiologists, biologists, epidemiologists and infectologists. Knowing thermodynamic properties and mechanistic models that are developed in biothermodynamics can shed more light on basic processes from the domains of biophysics and chemistry, which represent the basis for biological phenomena. The immune response (humoral) implies antigen–antibody reaction. The antigen–antibody reaction is similar to protein–ligand interactions. Thus, the driving force for the antigen–antibody interaction is the Gibbs energy of this interaction. Cellular immune response is related to mobilization of immune cells and, thus, increase in number. This results in growth. Growth, like with other cells, represents a biological and biothermodynamic phenomenon, driven by Gibbs energy of growth. Thus, it is necessary to know the thermodynamic properties of immune cells. After an extensive search of the literature, the author could not find data on the thermodynamic properties of lymphocytes, leukocytes and macrophages. Infection is a complex biological process, which, except for the infective agent (microorganism), involves a host cell/tissue and immune cells. To reveal the thermodynamic basis of infections in full, it is necessary to know all 3 elements (thermodynamic properties of microorganisms, immune system and host cells). Biothermodynamics is a young discipline and obviously faces many challenges and a great body of work that needs to be realized. The effort on the development of biothermodynamics seems justified since it can greatly help in explanation of pathogeneses of many diseases that occur as a result of microorganism–host interactions.

Time evolution of viruses can be followed through change in Gibbs energies of binding and biosynthesis [180]. Viruses exhibit a tendency to mutate. RNA viruses exhibit a greater tendency to mutate than DNA viruses [82]. Mutations lead to change in the sequence of nucleotides in nucleic acid, information contained in the virus nucleic acid, but also change in empirical formula of the virus and its thermodynamic properties, as well as the conformational change in the virus antigen. Change in one or several nucleotides during mutations leads to changes in one or several amino acids in the viral antigen, which in turn leads to change in elemental composition. Change in elemental composition leads to change in thermodynamic properties and conformational changes in the virus antigen. The changes that lead to more negative Gibbs energy give an advantage to the new virus strain. Mutations in viruses occur significantly more often than those that have caused pandemic waves. Many mutations have most likely proved themselves unsuccessful and such strains have disappeared from the population. This means that Gibbs energy of binding and biosynthesis, as well as conformational changes in the antigen, did not give an advantage (e.g., Gibbs energies of binding and biosynthesis became less negative). During time and acquisition of new mutations, it is possible to follow changes in thermodynamic properties of viruses. A tendency was observed in the temporal evolution of viruses towards more negative Gibbs energy of binding [180]. This can be related to the prediction of the theory of evolution that viruses increasingly adapt to their host with time [180].

## 4. Conclusions

Biothermodynamics of viruses is among the youngest scientific disciplines. However, appearance of new viruses, their rapid mutation, which can lead to epidemics and pandemics with a great number of cases and casualties, have given an impulse for the very rapid development of biothermodynamics of viruses. Knowing biothermodynamic properties can give useful information to epidemiologists and infectologists about the mechanism of virus–host interaction and virus–virus competition. Knowing empirical formulas of viruses is significant because it allows fast and accurate identification of known viruses or detection of new viruses or variants. Moreover, phenomenological equations, which belong to nonequilibrium thermodynamics, have proven themselves an important tool for analysis of rates of antigen–receptor binding and rates of virus multiplication inside host cells. The permissiveness coefficient could be useful during the estimation of the degree of damage to host tissues, caused by the multiplication of viruses, as well as the assessment of the outcome of virus–virus competition during the simultaneous presence of two viruses or virus variants in the same time and the same place.

## References

[1] Carnot L. (1786). Essai sur les Machines en Général.

[2] Carnot L. (1803). Principes Fondamentaux de l’Equilibre et du Movement.

[3] Carnot S. (1824). Réflexions sur la Puissance Motrice du Feu et sur les Machines Propres à Développer Cette Puissance.

[4] Müller I. (2010). A History of Thermodynamics: The Doctrine of Energy and Entropy.

[5] Lavoisier A.L., Marquis de Laplace P.S. (1783). Mémoire sur la Chaleur: Lû à l’Académie Royale des Sciences, le 28 Juin 1783.

[6] Lavoisier A.L., DeLaplace P.S. (1994). Memoir on heat read to the royal academy of sciences, 28 June 1783. Obes. Res..

[7] Boltzmann L., McGuinnes B. (1974). The second law of thermodynamics. Theoretical Physics and Philosophical Problems.

[8] Clausius R. (1867). The Mechanical Theory of Heat—With its Applications to the Steam Engine and to Physical Properties of Bodies.

[9] Clausius R. (1870). On a Mechanical Theorem Applicable to Heat. Philos. Mag. Ser..

[10] Clausius R., Kestin J. (1976). On different forms of the fundamental equations of the mechanical theory of heat and their convenience for application. The Second Law of Thermodynamics.

[11] Von Bertalanffy L. (1950). The theory of open systems in physics and biology. Science.

[12] Schrödinger E. (1944). What is Life? The Physical Aspect of the Living Cell.

[13] Morowitz H.J. (1992). Beginnings of Cellular Life: Metabolism Recapitulates Biogenesis.

[14] Morowitz H.J. (1968). Energy Flow in Biology: Biological Organization as a Problem in Thermal Physics.

[15] Morowitz H.J. (1955). Some order-disorder considerations in living systems. Bull. Math. Biophys..

[16] Von Stockar U., Liu J.-S. (1999). Does microbial life always feed on negative entropy? Thermodynamic analysis of microbial growth. Biochim. Biophys. Acta (BBA)—Bioenerg..

[17] Von Stockar U., von Stockar U. (2013). Live cells as open non-equilibrium systems. Biothermodynamics: The Role of Thermodynamics in Biochemical Engineering.

[18] Von Stockar U., von Stockar U. (2013). Biothermodynamics of live cells: Energy dissipation and heat generation in cellular structures. Biothermodynamics: The Role of Thermodynamics in Biochemical Engineering.

[19] Von Stockar U., Maskow T., Liu J., Marison I.W., Patiño R. (2006). Thermodynamics of microbial growth and metabolism: An analysis of the current situation. J. Biotechnol..

[20] Patiño R., Janssen M., von Stockar U. (2006). A study of the growth for the microalgaChlorella vulgaris by photo-bio-calorimetry and other on-line and off-line techniques. Biotechnol. Bioeng..

[21] Hansen L.D., Tolley H.D., Woodfield B.F. (2021). Transformation of matter in living organisms during growth and evolution. Biophys. Chem..

[22] Hansen L.D., Popovic M., Tolley H.D., Woodfield B.F. (2018). Laws of evolution parallel the laws of thermodynamics. J. Chem. Thermodyn..

[23] Hansen L.D., Criddle R.S., Battley E.H. (2009). Biological calorimetry and the thermodynamics of the origination and evolution of life. Pure Appl. Chem..

[24] Skene K.R. (2015). Life’s a Gas: A Thermodynamic Theory of Biological Evolution. Entropy.

[25] Battley E.H. (2013). A Theoretical Study of the Thermodynamics of Microbial Growth Using*Saccharomyces cerevisiae* and a Different Free Energy Equation. Q. Rev. Biol..

[26] Battley E.H. (1999). An empirical method for estimating the entropy of formation and the absolute entropy of dried microbial biomass for use in studies on the thermodynamics of microbial growth. Thermochim. Acta.

[27] Battley E.H., Kemp E.B. (1999). The thermodynamics of microbial growth. Handbook of Thermal Analysis and Calorimetry, vol. 4: From Macromolecules to Man.

[28] Battley E.H. (1998). The development of direct and indirect methods for the study of the thermodynamics of microbial growth. Thermochim. Acta.

[29] Battley E.H., Putnam R.L., Boerio-Goates J. (1997). Heat capacity measurements from 10 to 300 K and derived thermodynamic functions of lyophilized cells of Saccharomyces cerevisiae including the absolute entropy and the entropy of formation at 298.15 K. Thermochim. Acta.

[30] Battley E.H. (1992). On the enthalpy of formation ofEscherichia coli K-12 cells. Biotechnol. Bioeng..

[31] Roels J.A. (1983). Energetics and Kinetics in Biotechnology.

[32] Sandler S.I., Orbey H. (1991). On the thermodynamics of microbial growth processes. Biotechnol. Bioeng..

[33] Sandler S.I. (2017). Chemical, Biochemical, and Engineering Thermodynamics.

[34] Barros N. (2021). Thermodynamics of Soil Microbial Metabolism: Applications and Functions. Appl. Sci..

[35] Barros N., Fernandez I., Byrne K.A., Jovani-Sancho A.J., Ros-Mangriñan E., Hansen L.D. (2020). Thermodynamics of soil organic matter decomposition in semi-natural oak (*Quercus*) woodland in southwest Ireland. Oikos.

[36] Barros N., Hansen L., Piñeiro V., Pérez-Cruzado C., Villanueva M., Proupín J., Rodríguez-Añón J. (2016). Factors influencing the calorespirometric ratios of soil microbial metabolism. Soil Biol. Biochem..

[37] Maskow T., von Stockar U. (2013). Miniaturization of calorimetry: Strengths and weaknesses for bioprocess monitoring. Biothermodynamics: The Role of Thermodynamics in Biochemical Engineering.

[38] Maskow T., Harms H. (2006). Real Time Insights into Bioprocesses Using Calorimetry: State of the Art and Potential. Eng. Life Sci..

[39] Maskow T., Paufler S. (2015). What does calorimetry and thermodynamics of living cells tell us?. Methods.

[40] Maskow T., Kemp R.B., Buchholz F., Schubert T., Kiesel B., Harms H. (2009). What heat is telling us about microbial conversions in nature and technology: From chip- to megacalorimetry. Microb. Biotechnol..

[41] Maskow T., Kiesel B., Schubert T., Yong Z., Harms H., Yao J. (2010). Calorimetric real time monitoring of lambda prophage induction. J. Virol. Methods.

[42] Guosheng L., Yi L., Xiangdong C., Peng L., Ping S., Songsheng Q. (2003). Study on interaction between T4 phage and Escherichia coli B by microcalorimetric method. J. Virol. Methods.

[43] Von Stockar U., Maskow T., Vojinovic V., von Stockar U. (2013). Thermodynamic analysis of metabolic pathways. Biothermodynamics: The Role of Thermodynamics in Biochemical Engineering.

[44] Meurer F., Bobrownik M., Sadowski G., Held C. (2016). Standard Gibbs Energy of Metabolic Reactions: I. Hexokinase Reaction. Biochemistry.

[45] Meurer F., Do H.T., Sadowski G., Held C. (2017). Standard Gibbs energy of metabolic reactions: II. Glucose-6-phosphatase reaction and ATP hydrolysis. Biophys. Chem..

[46] Wangler A., Schmidt C., Sadowski G., Held C. (2018). Standard Gibbs Energy of Metabolic Reactions: III The 3-Phosphoglycerate Kinase Reaction. ACS Omega.

[47] Greinert T., Baumhove K., Sadowski G., Held C. (2020). Standard Gibbs energy of metabolic reactions: IV. Triosephosphate isomerase reaction. Biophys. Chem..

[48] Greinert T., Vogel K., Seifert A.I., Siewert R., Andreeva I.V., Verevkin S.P., Maskow T., Sadowski G., Held C. (2020). Standard Gibbs energy of metabolic reactions: V. Enolase reaction. Biochim. et Biophys. Acta (BBA)—Proteins Proteom..

[49] Greinert T., Vogel K., Mühlenweg J.-K., Sadowski G., Maskow T., Held C. (2020). Standard Gibbs energy of metabolic reactions: VI. Glyceraldehyde 3-phosphate dehydrogenase reaction. Fluid Phase Equilibria.

[50] Popovic M., Woodfield B.F., Hansen L.D. (2018). Thermodynamics of hydrolysis of cellulose to glucose from 0 to 100 °C: Cellulosic biofuel applications and climate change implications. J. Chem. Thermodyn..

[51] Niebel B., Leupold S., Heinemann M. (2019). An upper limit on Gibbs energy dissipation governs cellular metabolism. Nat. Metab..

[52] Ould-Moulaye C., Dussap C., Gros J. (1999). Estimation of Gibbs energy changes of central metabolism reactions. Biotechnol. Tech..

[53] Kümmel A., Panke S., Heinemann M. (2006). Systematic assignment of thermodynamic constraints in metabolic network models. BMC Bioinform..

[54] Berg J.M., Tymoczko J.L., Stryer L. (2002). Biochemistry.

[55] Annamalai K. (2021). Oxygen Deficient (OD) Combustion and Metabolism: Allometric Laws of Organs and Kleiber’s Law from OD Metabolism?. Systems.

[56] Annamalai K., Miller J.A. Link between O_2_ Deficient Metabolism in Organs and Group Combustion in Engineering. Proceedings of the 10th US National Combustion Meeting.

[57] Annamalai K., Nanda A. (2017). Biological Aging and Life Span Based on Entropy Stress via Organ and Mitochondrial Metabolic Loading. Entropy.

[58] Annamalai K., Silva C. (2012). Entropy Stress and Scaling of Vital Organs over Life Span Based on Allometric Laws. Entropy.

[59] Silva C.A., Annamalai K. (2009). Entropy Generation and Human Aging: Lifespan Entropy and Effect of Diet Composition and Caloric Restriction Diets. J. Thermodyn..

[60] Silva C., Annamalai K. (2008). Entropy Generation and Human Aging: Lifespan Entropy and Effect of Physical Activity Level. Entropy.

[61] Silva C., Annamalai K. 1st Law, Metabolism; 2nd Law and Entropy Generaion: Secret to Longevity in Lifespan?. Proceedings of the 9th AIAA/ASME Joint Thermophysics and Heat Transfer Conference.

[62] Hayflick L., Rattan S., Hayflick L. (2016). Unlike the Stochastic Events That Determine Ageing, Sex Determines Longevity. Cellular Ageing and Replicative Senescence. Healthy Ageing and Longevity.

[63] Hayflick L. (2010). Unlike ageing, longevity is sexually transmitted. Médecine Longévité.

[64] Hayflick L. (2007). Entropy Explains Aging, Genetic Determinism Explains Longevity, and Undefined Terminology Explains Misunderstanding Both. PLoS Genet..

[65] Hayflick L. (2007). Biological Aging Is No Longer an Unsolved Problem. Ann. N. York Acad. Sci..

[66] Hayflick L., Rattan S. (2003). Modulating aging, longevity determination and the diseases of old age. Modulating Aging and Longevity.

[67] Hayflick L. (2001). The Quest for Immortality: Science at the Frontiers of Aging. Radiat. Res..

[68] Hayflick L. (1998). How and why we age. Exp. Gerontol..

[69] Hayflick L. (1985). Theories of biological aging. Exp. Gerontol..

[70] Wu K.J. (2020). There Are More Viruses than Stars in the Universe. Why Do Only Some Infect Us?. National Geographic Magazine.

[71] Dance A. (2021). Beyond coronavirus: The virus discoveries transforming biology. Nature.

[72] Popovic M. (2022). Biothermodynamic Key Opens the Door of Life Sciences: Bridging the Gap between Biology and Thermodynamics. Preprints.

[73] Popovic M. (2017). Living organisms from Prigogine’s perspective: An opportunity to introduce students to biological entropy balance. J. Biol. Educ..

[74] Popovic M.E. (2018). Research in entropy wonterland: A review of the entropy concept. Therm. Sci..

[75] Popovic M. (2018). Researchers in an Entropy Wonderland: A Review of the Entropy Concept. Therm. Sci..

[76] Wimmer E. (2006). The test-tube synthesis of a chemical called poliovirus. EMBO Rep..

[77] Molla A., Paul A.V., Wimmer E. (1991). Cell-Free, De Novo Synthesis of Poliovirus. Science.

[78] Popovic M. (2022). Atom counting method for determining elemental composition of viruses and its applications in biothermodynamics and environmental science. Comput. Biol. Chem..

[79] Popovic M., Minceva M. (2020). A thermodynamic insight into viral infections: Do viruses in a lytic cycle hijack cell metabolism due to their low Gibbs energy?. Heliyon.

[80] Popovic M., Minceva M. (2020). Thermodynamic insight into viral infections 2: Empirical formulas, molecular compositions and thermodynamic properties of SARS, MERS and SARS-CoV-2 (COVID-19) viruses. Heliyon.

[81] Popovic M., Minceva M. (2021). Coinfection and Interference Phenomena Are the Results of Multiple Thermodynamic Competitive Interactions. Microorganisms.

[82] Duffy S. (2018). Why are RNA virus mutation rates so damn high?. PLoS Biol..

[83] Callaway E. (2020). The coronavirus is mutating—Does it matter?. Nature.

[84] Barton M.I., MacGowan S.A., Kutuzov M.A., Dushek O., Barton G.J., van der Merwe P.A. (2021). Effects of common mutations in the SARS-CoV-2 Spike RBD and its ligand, the human ACE2 receptor on binding affinity and kinetics. eLife.

[85] Wang R., Chen J., Gao K., Hozumi Y., Yin C., Wei G.-W. (2021). Analysis of SARS-CoV-2 mutations in the United States suggests presence of four substrains and novel variants. Commun. Biol..

[86] Popovic M., Popovic M. (2022). Strain Wars: Competitive interactions between SARS-CoV-2 strains are explained by Gibbs energy of antigen-receptor binding. Microb. Risk Anal..

[87] Popovic M. (2022). Strain wars 2: Binding constants, enthalpies, entropies, Gibbs energies and rates of binding of SARS-CoV-2 variants. Virology.

[88] Popovic M. (2022). Strain wars 3: Differences in infectivity and pathogenicity between Delta and Omicron strains of SARS-CoV-2 can be explained by thermodynamic and kinetic parameters of binding and growth. Microb. Risk Anal..

[89] Popovic M. (2022). Strain wars 4—Darwinian evolution through Gibbs’ glasses: Gibbs energies of binding and growth explain evolution of SARS-CoV-2 from Hu-1 to BA.2. Virology.

[90] Popovic M. (2022). Strain wars 5: Gibbs energies of binding of BA.1 through BA.4 variants of SARS-CoV-2. Microb. Risk Anal..

[91] Popovic M. (2022). Omicron BA.2.75 Subvariant of SARS-CoV-2 Is Expected to Have the Greatest Infectivity Compared with the Competing BA.2 and BA.5, Due to Most Negative Gibbs Energy of Binding. BioTech.

[92] Şimşek B., Özilgen M., Utku F. (2021). How much energy is stored in SARS-CoV-2 and its structural elements?. Energy Storage.

[93] Degueldre C. (2021). Single virus inductively coupled plasma mass spectroscopy analysis: A comprehensive study. Talanta.

[94] Popovic M. (2022). Formulas for death and life: Chemical composition and biothermodynamic properties of Monkeypox (MPV, MPXV, HMPXV) and Vaccinia (VACV) viruses. Therm. Sci..

[95] Popovic M. (2022). Why doesn’t Ebola virus cause pandemics like SARS-CoV-2?. Microb. Risk Anal..

[96] Du X., Li Y., Xia Y.-L., Ai S.-M., Liang J., Sang P., Ji X.-L., Liu S.-Q. (2016). Insights into Protein–Ligand Interactions: Mechanisms, Models, and Methods. Int. J. Mol. Sci..

[97] Popovic M. (2022). Standard Gibbs Energy of Binding of the gp120 Antigen of HIV-1 to the CD4 Receptor. Preprints.

[98] Gale P. (2021). Using thermodynamic equilibrium models to predict the effect of antiviral agents on infectivity: Theoretical application to SARS-CoV-2 and other viruses. Microb. Risk Anal..

[99] Gale P. (2020). How virus size and attachment parameters affect the temperature sensitivity of virus binding to host cells: Predictions of a thermodynamic model for arboviruses and HIV. Microb. Risk Anal..

[100] Gale P. (2019). Towards a thermodynamic mechanistic model for the effect of temperature on arthropod vector competence for transmission of arboviruses. Microb. Risk Anal..

[101] Gale P. (2018). Using thermodynamic parameters to calibrate a mechanistic dose-response for infection of a host by a virus. Microb. Risk Anal..

[102] Lucia U., Grisolia G., Deisboeck T.S. (2020). Seebeck-like effect in SARS-CoV-2 Bio-thermodynamics. Atti Accad. Peloritana Pericolanti.

[103] Lucia U., Deisboeck T.S., Grisolia G. (2020). Entropy-Based Pandemics Forecasting. Front. Phys..

[104] Casasnovas J.M., Springer T.A. (1995). Kinetics and Thermodynamics of Virus Binding to Receptor. J. Biol. Chem..

[105] Popovic M.E., Minceva M. (2020). Thermodynamic properties of human tissues. Therm. Sci..

[106] Popovic M., Minceva M. (2021). Standard Thermodynamic Properties, Biosynthesis Rates, and the Driving Force of Growth of Five Agricultural Plants. Front. Plant Sci..

[107] Popovic M. (2019). Thermodynamic properties of microorganisms: Determination and analysis of enthalpy, entropy, and Gibbs free energy of biomass, cells and colonies of 32 microorganism species. Heliyon.

[108] Popovic M., Stenning G.B., Göttlein A., Minceva M. (2021). Elemental composition, heat capacity from 2 to 300 K and derived thermodynamic functions of 5 microorganism species. J. Biotechnol..

[109] Duboc P., Marison I., Von Stockar U., Kemp E.B. (1999). Quantitative calorimetry and biochemical engineering. Handbook of Thermal Analysis and Calorimetry, vol. 4: From Macromolecules to Man.

[110] Wang H.Y., Mou D.-G., Swartz J.R. (1976). Thermodynamic evaluation of microbial growth. Biotechnol. Bioeng..

[111] Hou W., Armstrong N., Obwolo L.A., Thomas M., Pang X., Jones K.S., Tang Q. (2017). Determination of the Cell Permissiveness Spectrum, Mode of RNA Replication, and RNA-Protein Interaction of Zika Virus. BMC Infect. Dis..

[112] Popovic M. (2022). Omicron BA.2.75 Sublineage (Centaurus) Follows the Expectations of the Evolution Theory: Less Negative Gibbs Energy of Biosynthesis Indicates Decreased Pathogenicity. Microbiol. Res..

[113] Buzón P., Maity S., Roos W.H. (2020). Physical virology: From virus self-assembly to particle mechanics. WIREs Nanomed. Nanobiotechnology.

[114] Garmann R.F., Goldfain A.M., Manoharan V.N. (2019). Measurements of the self-assembly kinetics of individual viral capsids around their RNA genome. Proc. Natl. Acad. Sci. USA.

[115] Popovic M. (2014). Comparative study of entropy and information change in closed and open thermodynamic systems. Thermochim. Acta.

[116] Popovic M. (2014). Entropy change of open thermodynamic systems in self-organizing processes. Therm. Sci..

[117] Popovic M.E., Minceva M. (2021). Comment on: “A critical review on heat and mass transfer modelling of viral infection and virion evolution: The case of SARS-COV2”. Therm. Sci..

[118] Rusnati M., Chiodelli P., Bugatti A., Urbinati C. (2013). Bridging the past and the future of virology: Surface plasmon resonance as a powerful tool to investigate virus/host interactions. Crit. Rev. Microbiol..

[119] Han P., Li L., Liu S., Wang Q., Zhang D., Xu Z., Han P., Li X., Peng Q., Su C. (2022). Receptor binding and complex structures of human ACE2 to spike RBD from omicron and delta SARS-CoV-2. Cell.

[120] Beatty J., Beatty B.G., Vlahos W.G. (1987). Measurement of monoclonal antibody affinity by non-competitive enzyme immunoassay. J. Immunol. Methods.

[121] Wu L., Zhou L., Mo M., Liu T., Wu C., Gong C., Lu K., Gong L., Zhu W., Xu Z. (2022). SARS-CoV-2 Omicron RBD shows weaker binding affinity than the currently dominant Delta variant to human ACE2. Signal Transduct. Target. Ther..

[122] Privalov P.L. (2012). Microcalorimetry of Macromolecules: The Physical Basis of Biological Structures.

[123] Sarge S.M., Höhne G.W., Hemminger W. (2014). Calorimetry: Fundamentals, Instrumentation and Applications.

[124] Manin C., Krell T., Nicolaï M.-C., Pierre-Justin C., Bérard Y., Brass O., Gérentes L., Leung-Tack P., Chevalier M. (2005). Characterization of different strains of poliovirus and influenza virus by differential scanning calorimetry. Biotechnol. Appl. Biochem..

[125] Yang Y., Zhao Q., Li Z., Sun L., Ma G., Zhang S., Su Z. (2017). Stabilization study of inactivated foot and mouth disease virus vaccine by size-exclusion HPLC and differential scanning calorimetry. Vaccine.

[126] Bauer D.W., Li D., Huffman J., Homa F.L., Wilson K., Leavitt J.C., Casjens S.R., Baines J., Evilevitch A. (2015). Exploring the Balance between DNA Pressure and Capsid Stability in Herpesviruses and Phages. J. Virol..

[127] Bauer D.W., Huffman J.B., Homa F.L., Evilevitch A. (2013). Herpes Virus Genome, The Pressure Is On. J. Am. Chem. Soc..

[128] Makarov V.V., Skurat E., Semenyuk P., Abashkin D.A., Kalinina N.O., Arutyunyan A.M., Solovyev A.G., Dobrov E.N. (2013). Structural Lability of Barley Stripe Mosaic Virus Virions. PLoS ONE.

[129] Virudachalam R., Harrington M., Markley J.L. (1985). Thermal stability of cowpea mosaic virus components: Differential scanning calorimetry studies. Virology.

[130] Virudachalam R., Low P.S., Argos P., Markley J.L. (1985). Turnip yellow mosaic virus and its capsid have thermal stabilities with opposite ph dependence: Studies by differential scanning calorimetry and 31P nuclear magnetic resonance spectroscopy. Virology.

[131] Toinon A., Greco F., Moreno N., Nicolai M.C., Guinet-Morlot F., Manin C., Ronzon F. (2015). Study of rabies virus by Differential Scanning Calorimetry. Biochem. Biophys. Rep..

[132] Brouillette C., Compans R., Brandts J., Segrest J. (1982). Structural domains of vesicular stomatitis virus. A study by differential scanning calorimetry, thermal gel analysis, and thermal electron microscopy. J. Biol. Chem..

[133] Banerjee M., Speir J.A., Kwan M.H., Huang R., Aryanpur P.P., Bothner B., Johnson J.E. (2010). Structure and Function of a Genetically Engineered Mimic of a Nonenveloped Virus Entry Intermediate. J. Virol..

[134] Nebel S., Bartoldus I., Stegmann T. (1995). Calorimetric detection of influenza virus induced membrane fusion. Biochemistry.

[135] Sturtevant J.M., Velicelebi G., Jaenicke R., Lauffer M.A. (1981). Scanning calorimetric investigation of the polymerization of the coat protein of tobacco mosaic virus. Biochemistry.

[136] Stauffer H., Srinivasan S., Lauffer M.A. (1970). Calorimetric studies on polymerization-depolymerization of tobacco mosaic virus protein. XIII. Biochemistry.

[137] Deschuyteneer M., Elouahabi A., Plainchamp D., Plisnier M., Soete D., Corazza Y., Lockman L., Giannini S., Deschamps M. (2010). Molecular and structural characterization of the L1 virus-like particles that are used as vaccine antigens in*Cervarix*™, the AS04-adjuvanted HPV-16 and -18 cervical cancer vaccine. Hum. Vaccines.

[138] Wang G., Wang H.-J., Zhou H., Nian Q.-G., Song Z., Deng Y.-Q., Wang X., Zhu S.-Y., Li X.-F., Qin C.-F. (2015). Hydrated Silica Exterior Produced by Biomimetic Silicification Confers Viral Vaccine Heat-Resistance. ACS Nano.

[139] Yu M., Zhang S., Zhang Y., Yang Y., Ma G., Su Z. (2015). Microcalorimetric study of adsorption and disassembling of virus-like particles on anion exchange chromatography media. J. Chromatogr. A.

[140] Javorsky A., Maddumage J.C., Mackie E.R.R., da Costa T.P.S., Humbert P.O., Kvansakul M. (2022). Structural insight into the Scribble PDZ domains interaction with the oncogenic Human T-cell lymphotrophic virus-1 (HTLV-1) Tax1 PBM. FEBS J..

[141] Prins K.C., Binning J.M., Shabman R.S., Leung D.W., Amarasinghe G.K., Basler C.F. (2010). Basic Residues within the Ebolavirus VP35 Protein Are Required for Its Viral Polymerase Cofactor Function. J. Virol..

[142] Anasir M.I., Caria S., Skinner M.A., Kvansakul M. (2017). Structural basis of apoptosis inhibition by the fowlpox virus protein FPV039. J. Biol. Chem..

[143] Aladag A., Hoffmann S., Stoldt M., Bösing C., Willbold D., Schwarten M. (2014). Hepatitis C virus NS5A is able to competitively displace c-Myc from the Bin1 SH3 domain in vitro. J. Pept. Sci..

[144] Liu T., Sae-Ueng U., Li D., Lander G.C., Zuo X., Jönsson B., Rau D., Shefer I., Evilevitch A. (2014). Solid-to-fluid–like DNA transition in viruses facilitates infection. Proc. Natl. Acad. Sci. USA.

[145] Maassen S.J., Huskens J., Cornelissen J.J.L.M. (2019). Elucidating the Thermodynamic Driving Forces of Polyanion-Templated Virus-like Particle Assembly. J. Phys. Chem. B.

[146] Yang Y., Song Y., Lin X., Li S., Li Z., Zhao Q., Ma G., Zhang S., Su Z. (2020). Mechanism of bio-macromolecule denaturation on solid-liquid surface of ion-exchange chromatographic media—A case study for inactivated foot-and-mouth disease virus. J. Chromatogr. B.

[147] Kawahara T., Akiba I., Sakou M., Sakaguchi T., Taniguchi H. (2018). Inactivation of human and avian influenza viruses by potassium oleate of natural soap component through exothermic interaction. PLoS ONE.

[148] Gao X., Zhu K., Qin B., Olieric V., Wang M., Cui S. (2021). Crystal structure of SARS-CoV-2 Orf9b in complex with human TOM70 suggests unusual virus-host interactions. Nat. Commun..

[149] Zhou J., Rong X.-L., Cao X., Tang Q., Liu D., Jin Y.-H., Shi X.-X., Zhong M., Zhao Y., Yang Y. (2022). Assembly of Poly(ethylene glycol)ylated Oleanolic Acid on a Linear Polymer as a Pseudomucin for Influenza Virus Inhibition and Adsorption. Biomacromolecules.

[150] Noble C.G., Lim S.P., Arora R., Yokokawa F., Nilar S., Seh C.C., Wright S.K., Benson T.E., Smith P.W., Shi P.-Y. (2016). A Conserved Pocket in the Dengue Virus Polymerase Identified through Fragment-based Screening. J. Biol. Chem..

[151] Sharma R., Fatma B., Saha A., Bajpai S., Sistla S., Dash P.K., Parida M., Kumar P., Tomar S. (2016). Inhibition of chikungunya virus by picolinate that targets viral capsid protein. Virology.

[152] Byrn R.A., Jones S.M., Bennett H.B., Bral C., Clark M.P., Jacobs M.D., Kwong A.D., Ledeboer M.W., Leeman J.R., McNeil C.F. (2015). Preclinical Activity of VX-787, a First-in-Class, Orally Bioavailable Inhibitor of the Influenza Virus Polymerase PB2 Subunit. Antimicrob. Agents Chemother..

[153] Vorobieva N., Sanina N., Vorontsov V., Kostetsky E., Mazeika A., Tsybulsky A., Kim N., Shnyrov V. (2014). On the possibility of lipid-induced regulation of conformation and immunogenicity of influenza a virus H1/N1 hemagglutinin as antigen of TI-complexes. J. Mol. Microbiol. Biotechnol..

[154] Sigg A.P., Mariotti M., Grütter A.E., Lafranca T., Leitner L., Bonkat G., Braissant O. (2022). A Method to Determine the Efficacy of a Commercial Phage Preparation against Uropathogens in Urine and Artificial Urine Determined by Isothermal Microcalorimetry. Microorganisms.

[155] Tkhilaishvili T., Di Luca M., Abbandonato G., Maiolo E.M., Klatt A.-B., Reuter M., Möncke-Buchner E., Trampuz A. (2018). Real-time assessment of bacteriophage T3-derived antimicrobial activity against planktonic and biofilm-embedded Escherichia coli by isothermal microcalorimetry. Res. Microbiol..

[156] Morais F.M., Buchholz F., Hartmann T., Lerchner J., Neu T., Kiesel B., Harms H., Maskow T. (2013). Chip-calorimetric monitoring of biofilm eradication with bacteriophages reveals an unexpected infection-related heat profile. J. Therm. Anal..

[157] Tkhilaishvili T., Wang L., Tavanti A., Trampuz A., Di Luca M. (2020). Antibacterial Efficacy of Two Commercially Available Bacteriophage Formulations, Staphylococcal Bacteriophage and PYO Bacteriophage, Against Methicillin-Resistant Staphylococcus aureus: Prevention and Eradication of Biofilm Formation and Control of a Systemic Infection of Galleria mellonella Larvae. Front. Microbiol..

[158] Tkhilaishvili T., Wang L., Perka C., Trampuz A., Moreno M.G. (2020). Using Bacteriophages as a Trojan Horse to the Killing of Dual-Species Biofilm Formed by Pseudomonas aeruginosa and Methicillin Resistant Staphylococcus aureus. Front. Microbiol..

[159] Tkhilaishvili T., Lombardi L., Klatt A.-B., Trampuz A., Di Luca M. (2018). Bacteriophage Sb-1 enhances antibiotic activity against biofilm, degrades exopolysaccharide matrix and targets persisters of Staphylococcus aureus. Int. J. Antimicrob. Agents.

[160] Tkhilaishvili T., Di Luca M., Trampuz A. (2018). Simultaneous and sequential applications of phages and ciprofloxacin in killing mixed-species biofilm of pseudomonas aeruginosa and staphylococcus aureus. Orthopaedic Proceedings.

[161] Wang L., Tkhilaishvili T., Trampuz A. (2020). Adjunctive Use of Phage Sb-1 in Antibiotics Enhances Inhibitory Biofilm Growth Activity versus Rifampin-Resistant *Staphylococcus aureus* Strains. Antibiotics.

[162] Wang L., Tkhilaishvili T., Trampuz A., Moreno M.G. (2020). Evaluation of Staphylococcal Bacteriophage Sb-1 as an Adjunctive Agent to Antibiotics Against Rifampin-Resistant Staphylococcus aureus Biofilms. Front. Microbiol..

[163] Pirlar R.F., Wagemans J., Benavente L.P., Lavigne R., Trampuz A., Moreno M.G. (2022). Novel Bacteriophage Specific against *Staphylococcus epidermidis* and with Antibiofilm Activity. Viruses.

[164] Wang L., Tkhilaishvili T., Bernal Andres B., Trampuz A., Gonzalez Moreno M. (2020). Bacteriophage–antibiotic combinations against ciprofloxacin/ceftriaxone-resistant Escherichia coli in vitro and in an experimental Galleria mellonella model. Int. J. Antimicrob. Agents.

[165] Shadrick W.R., Mukherjee S., Hanson A.M., Sweeney N.L., Frick D.N. (2013). Aurintricarboxylic Acid Modulates the Affinity of Hepatitis C Virus NS3 Helicase for Both Nucleic Acid and ATP. Biochemistry.

[166] Tkhilaishvili T. (2022). Bacteriophages as an Alternative Strategy in the Treatment and Prevention of Implant-Associated Infections. Ph.D. Thesis.

[167] Gelman D., Yerushalmy O., Alkalay-Oren S., Rakov C., Ben-Porat S., Khalifa L., Adler K., Abdalrhman M., Coppenhagen-Glazer S., Aslam S. (2021). Clinical Phage Microbiology: A suggested framework and recommendations for the in-vitro matching steps of phage therapy. Lancet Microbe.

[168] Djamali E., Nulton J.D., Turner P.J., Rohwer F., Salamon P. (2012). Heat output by marine microbial and viral communities. J. Non-Equilibrium Thermodyn..

[169] Sayers E.W., Bolton E.E., Brister J.R., Canese K., Chan J., Comeau D.C., Connor R., Funk K., Kelly C., Kim S. (2021). Database resources of the national center for biotechnology information. Nucleic Acids Res..

[170] NCBI (2022). NCBI Database [online] National Center for Biotechnology Information. https://www.ncbi.nlm.nih.gov/.

[171] Khare S., Gurry C., Freitas L., Schultz M.B., Bach G., Diallo A., Akite N., Ho J., Lee R.T., Yeo W. (2021). GISAID’s Role in Pandemic Response. China CDC Wkly..

[172] Elbe S., Buckland-Merrett G. (2017). Data, disease and diplomacy: GISAID’s innovative contribution to global health. Glob. Chall..

[173] Shu Y., McCauley J. (2017). GISAID: Global initiative on sharing all influenza data—From vision to reality. Eurosurveillance.

[174] The UniProt Consortium (2021). UniProt: The universal protein knowledgebase in 2021. Nucleic Acids Res..

[175] Patel S.A., Erickson L.E. (1981). Estimation of heats of combustion of biomass from elemental analysis using available electron concepts. Biotechnol. Bioeng..

[176] Hurst J.E., Harrison B.K. (1992). Estimation of liquid and solid heat capacities using a modified kopp’s rule. Chem. Eng. Commun..

[177] Ozilgen M., Sorgüven E. (2017). Biothermodynamics: Principles and Applications.

[178] Demirel Y. (2014). Nonequilibrium Thermodynamics: Transport and Rate Processes in Physical, Chemical and Biological Systems.

[179] Balmer R.T. (2010). Modern Engineering Thermodynamics.

[180] Popovic M. (2022). Beyond COVID-19: Do biothermodynamic properties allow predicting the future evolution of SARS-CoV-2 variants?. Microb. Risk Anal..

[181] Olotu F.A., Agoni C., Soremekun O., Soliman M.E.S. (2020). The recent application of 3D-QSAR and docking studies to novel HIV-protease inhibitor drug discovery. Expert Opin. Drug Discov..

[182] Dănăilă V.-R., Avram S., Buiu C. (2022). The applications of machine learning in HIV neutralizing antibodies research—A systematic review. Artif. Intell. Med..

[183] Tu J., Li J.J., Shan Z.J., Zhai H.L. (2017). Exploring the binding mechanism of Heteroaryldihydropyrimidines and Hepatitis B Virus capsid combined 3D-QSAR and molecular dynamics. Antivir. Res..

[184] Singh A., Dauzhenka T., Kundrotas P.J., Sternberg M.J.E., Vakser I.A. (2020). Application of docking methodologies to modeled proteins. Proteins: Struct. Funct. Bioinform..

[185] Shahhosseini N., Babuadze G., Wong G., Kobinger G.P. (2021). Mutation Signatures and In Silico Docking of Novel SARS-CoV-2 Variants of Concern. Microorganisms.

[186] Gopi P., Gurnani M., Singh S., Sharma P., Pandya P. (2022). Structural aspects of SARS-CoV-2 mutations: Implications to plausible infectivity with ACE-2 using computational modeling approach. J. Biomol. Struct. Dyn..

[187] Park T., Lee S.Y., Kim S., Kim M.J., Kim H.G., Jun S., Kim S., Kim B.T., Park E.C., Park D. (2020). Spike protein binding prediction with neutralizing antibodies of SARS-CoV-2. BioRxiv.

[188] Schoeder C.T., Schmitz S., Adolf-Bryfogle J., Sevy A.M., Finn J.A., Sauer M.F., Bozhanova N.G., Mueller B.K., Sangha A.K., Bonet J. (2021). Modeling Immunity with Rosetta: Methods for Antibody and Antigen Design. Biochemistry.

[189] Atkins P.W., de Paula J. (2011). Physical Chemistry for the Life Sciences.

[190] Atkins P.W., de Paula J. (2014). Physical Chemistry: Thermodynamics, Structure, and Change.

[191] Liu J.-S., Vojinović V., Patiño R., Maskow T., von Stockar U. (2007). A comparison of various Gibbs energy dissipation correlations for predicting microbial growth yields. Thermochim. Acta.

[192] Gelderblom H.R., Baron S. (1996). Structure and Classification of Viruses. Medical Microbiology.

[193] Lucia U., Grisolia G., Deisboeck T.S. (2021). Thermodynamics and SARS-CoV-2: Neurological effects in post-Covid 19 syndrome. Atti Accad. Peloritana Pericolanti.

[194] Pinheiro A.V., Baptista P., Lima J.C. (2008). Light activation of transcription: Photocaging of nucleotides for control over RNA polymerization. Nucleic Acids Res..

[195] Lee J., Schwarz K.J., Kim D.S., Moore J.S., Jewett M.C. (2020). Ribosome-mediated polymerization of long chain carbon and cyclic amino acids into peptides in vitro. Nat. Commun..

[196] Dodd T., Botto M., Paul F., Fernandez-Leiro R., Lamers M.H., Ivanov I. (2020). Polymerization and editing modes of a high-fidelity DNA polymerase are linked by a well-defined path. Nat. Commun..

[197] Johansson E., Dixon N. (2013). Replicative DNA Polymerases. Cold Spring Harb. Perspect. Biol..

[198] Tallarida R.J. (2011). Quantitative Methods for Assessing Drug Synergism. Genes Cancer.

